# Association of Six Well-Characterized Polymorphisms in TNF-α and TNF-β Genes with Sarcoidosis: A Meta-Analysis

**DOI:** 10.1371/journal.pone.0080150

**Published:** 2013-11-07

**Authors:** Yun Feng, Jianping Zhou, Chaohao Gu, Yongjie Ding, Huanying Wan, Lei Ni, Wenquan Niu

**Affiliations:** 1 Department of Respiration, Ruijin Hospital, School of Medicine, Shanghai Jiao Tong University, Shanghai, China; 2 College of Computer Science, Sichuan University, Chengdu, China; 3 State Key Laboratory of Medical Genomics, Ruijin Hospital, School of Medicine, Shanghai Jiao Tong University, Shanghai, China; University Hospital Freiburg, Germany

## Abstract

**Backgrounds:**

In this study, we aimed to investigate the association of six well-characterized polymorphisms in tumor necrosis factor alpha and beta (TNF-α and TNF-β) genes with the risk for sarcoidosis via a comprehensive meta-analysis.

**Methods And Findings:**

The electronic MEDLINE (Ovid) and PubMed databases covering the period from the earliest possible year to June 2013 were searched. Total 13 qualified articles including 1584 patients with sarcoidosis and 2636 controls were recruited. The data were analyzed by RevMan software, and risk estimates were expressed as odds ratios (ORs) and 95% confidence intervals (95% CIs). Analyses of the full data set failed to identify any significant association of TNF-α gene -307A (OR=1.25; 95% CI: 0.98-1.59), -1031C (OR=0.88; 95% CI: 0.71-1.1), -863A (OR=0.89; 95% CI: 0.72-1.11), -238A (OR=0.97; 95% CI: 0.71-1.32), and -857T (OR=1.14; 95% CI: 0.74-1.77) alleles, but a significant association for TNF-β 252A allele (OR=1.65; 95%CI = 1.33-2.04; P<0.00001). Under a random-effects allelic model, there was marginally significant increased risk of sarcoidosis for -307A allele among Caucasians (OR=1.25; 95% CI: 0.96-1.62; P=0.09) but not among Asians (OR=2.12; 95% CI: 0.31-14.27; P=0.44). There was a low probability of publication bias as reflected by the fail-safe number.

**Conclusions:**

This meta-analysis extended previous findings on the association between the TNF-α and TNF-β genetic polymorphisms and sarcoidosis, by showing that the TNF-β gene A252G polymorphism might be a potential risk factor for the development of sarcoidosis.

## Introduction

Sarcoidosis is a multiorgan inflammatory disorder with unknown etiology; it is characterized by the accumulated activation of the CD4+ T lymphocytes and macrophages at disease sites and by the formation of noncaseating epithelioid cell granulomas [1]. The onset age of sarcoidosis ranges from 20 to 40 years, and it often affects the lungs with 5% of the patients resulting in lung fibrosis, and eventually respiratory insufficiency [2,3]. Due to a significant but unpredictable risk of recurrence, sarcoidosis is suggested to be an independent clinical disease with a constitutional, and possibly a genetic component in its etiology. Muller-Quernheim and coworkers have written an excellent review on the genetic aspects of sarcoidosis [4]; however, to determine how many genes and which genetic determinants are actually involved in the pathogenesis of sarcoidosis so far remains an interpretive challenge.

Epidemiological data supported that the increased production of tumor necrosis factor (TNF) family members may have physiological implications on the development of sarcoidosis [5]. TNF family is a growing group of cytokines, and mainly includes TNF alpha and beta (TNF-α and TNF-β). The genes encoding TNF-α and TNF-β are located adjacent to each other in the major histocompatibility complex class III region on chromosome 6p21.3. Given the highly polymorphic sequences of both genes, it is of interest to determine which genetic defects have the causal potentials to regulate plasma TNF-α and TNF-β levels, further to precipitate the occurrence of sarcoidosis. Although exhaustive association studies have been undertaken to address this issue, no definitive conclusion has yet been reached in the literature, with irreproducible results. As a caveat, this lack of reproducibility might be mainly attributed to the ethnicity-specific genetic profiles and the individual underpowered studies. To generate more information, we carried out a meta-analysis of all available case-control studies to investigate the association of genetic polymorphisms of TNF-α and TNF-β with the risk of sarcoidosis. Selection of polymorphisms under study is straightforward if three or more unduplicated studies are available for a certain polymorphism in TNF-α and TNF-β genes.

## Methods

Meta-analysis of observational studies poses particular challenges due to the inherent biases and differences in study designs. In this context, we carried out this meta-analysis in accordance with the standards set forth by the Preferred Reporting Items for Systematic Reviews and Meta-analyses (PRISMA) guideline (please see Checklist S1) [6].

### Literature search

The electronic Medline (Ovid) and PubMed databases covering the period from the earliest possible year to June 2013 were searched for potentially eligible publications. The key words used for the search were “TNF-α or *TNFA* or TNF-β or lymphotoxin-α or *LTA*” and “sarcoidosis”, combined with “gene or variant or polymorphism or allele”. The research was also supplemented by reviews of reference lists, hand-searching of relevant journals, and correspondence with authors. If multiple publications were available from the same study group, the most complete and the recent results were abstracted. Search results were limited to articles published in English language and studies performed in human subjects.

### Inclusion/exclusion criteria

Our analyses were restricted to articles that fulfilled the following inclusion criteria (all must be satisfied): 1) if they investigated the association between genetic polymorphisms in TNF-α or TNF-β gene and sarcoidosis among unrelated subjects; 2) if they had genotypes of examined polymorphisms tested by validated sample size; 3) if they were on a case-control study design; 4) if they provided sufficient information on the genotypes or alleles of examined polymorphisms to allow an estimation of odds ratio (OR) and its corresponding 95% confidence interval (95% CI).

Articles were excluded (one was sufficient) if they investigated the progression, severity, phenotype modification, response to treatment or survival, as well as if they were conference abstracts, case reports/series, editorials, review articles, or the non-English articles.

### Data extraction

Data were extracted from qualified articles independently by two investigators (YF and WN) according to a standardized Excel template (Microsoft Corp, Redmond, WA). The following data were collected from each study: first author, year of publication, ethnicity, baseline characteristics of the study population, total number of cases and controls, and genotype or allele distributions in cases and controls. Information on the Hardy-Weinberg equilibrium test was also tracked or calculated if unavailable. After data extraction, discrepancies were adjudicated by discussion and a consensus was reached.

### Statistical analyses

The meta-analysis was calculated using the Review Manager version 5.0.19 software available at website http://ims.cochrane.org/revman/download. Hardy-Weinberg equilibrium was assessed with the Pearson χ^2^ test or the Fisher’s exact test (SAS version 9.1.3, Institute Inc., Cary, NC, USA).

The inconsistency index (*I* ^2^) was used to quantify the presence of between-study heterogeneity with a statistical significance of 0.1. In this study, we applied the random-effects model for all comparisons because it accommodates the possibility that the underlying effect differs across studies. For practical use, the random-effects model is more conservative and has a wider 95% CI than the fixed-effects model.

Sensitivity analyses were performed to look at more narrowly drawn subsets of the studies by removing an individual study each time or studies with similar features such as deviation from Hardy-Weinberg equilibrium to assess their separate influence. Predefined subgroup analyses were performed a priori according to the ethnicity.

Publication bias was assessed by the fail-safe number (*N*_fs_) with the significance set at 0.05 for each meta-comparison. Specifically, if the calculated *N*_fs_ value was smaller than the number of studies observed, the meta-analysis results might have publication bias. We calculated the *N*_fs0.05_ according to the formula *N*_fs0.05_ = (ΣZ/1.64) ^2^ - k, where *k* is the number of articles included.

## Results

### Study characteristics

**Figure 1:**
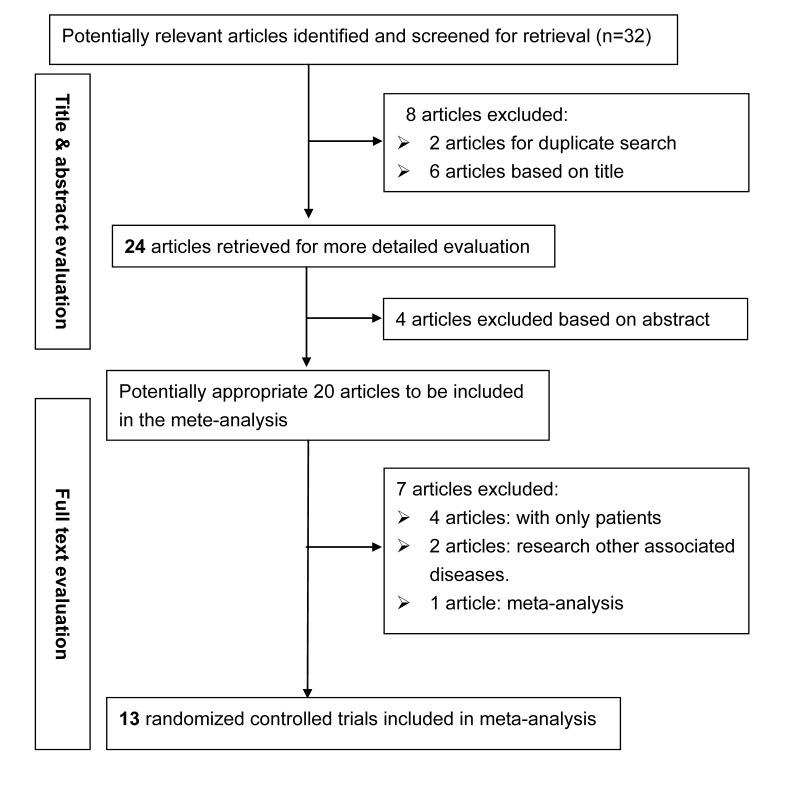
Flow diagram of search strategy and study selection.

Based on the search strategy, our primary search produced 32 potentially relevant articles, of which 13 qualified articles met the inclusion criteria [7-19]. In total, 1584 patients with sarcoidosis and 2636 controls and were examined. The detailed selection process is presented in Figure 1. The baseline characteristics of qualified studies are presented in Table 1. Of the 12 case-controls articles examining the association between the TNF-α gene G-307A polymorphism and sarcoidosis risk, 9 involved Caucasian populations [7,9,11-13,15-17,19], and 3 included Asian populations [8,10,14].

**Table 1:** The baseline characteristics of all study populations in the meta-analysis.

Polymorphism and Author	Year	Ethnicity	Total sample size		Allele distributions				Characteristics	
			Controls	Cases	Controls		Cases		
**TNF-α C-857T**					**C**	**T**	**C**	**T**		
Grutters JC et al.	2002	Caucasian	576	196	1068	84	339	53	The controls were sex- and age-matched healthy volunteers.	
Seyhan EC et al.	2008	Turkish	110	90	164	56	142	38	Controls (age: 45.39±13.1, M/F: 36/74). Cases (age: 44.5±11.2, M/F: 35/55).	
Sharma S et al.	2008	Indian	155	96	276	34	171	21	Cases (age: 43.5 9). Controls were age-matched healthy volunteers.	
Makrythanasis P et al.	2010	Greek	178	100	265	91	148	52	Controls (age: 30.7 ± 5.7). Cases (age: 53.3 ± 13.5).	
**TNF-α G307 A**					**G**	**A**	**G**	**A**		
Seitzer U et al.	1997	Germany	216	101	349	83	155	47	The controls were sex- and age-matched healthy volunteers.	
Takashige N et al.	1999	Japanese	125	26	248	2	47	5	The controls were sex- and age-matched healthy volunteers.	
Somoskövi A et al	1999	Germany	216	43	349	83	68	18	The controls were sex- and age-matched healthy volunteers.	
Yamaguchi E et al.	2001	Japanese	161	110	320	2	218	2	Cases (age: 39.4; M/F: 36/74). Controls were age-matched healthy volunteers.	
Labunski S et al.	2001	Germany	12	10	21	3	3	17	Cases (sarcoidosis and erythema nododsum patients). Controls were age-matched healthy volunteers.	
Pandey JP et al. (C)	2002	Caucasian	278	278	462	94	450	106	The controls were sex- and age-matched healthy volunteers.	
Pandey JP et al. (AA)	2002	African Americans	219	219	378	60	379	59	The controls were sex- and age-matched healthy volunteers.	
Grutters JC et al.	2002	Caucasian	576	196	927	225	316	76	The controls were sex- and age-matched healthy volunteers.	
Mrazek F et al.	2005	Czechs	232	114	381	83	173	55	Controls (age: 33.8±8.2); Cases (age: 44.8 ± 11.2).	
Sharma S et al.	2008	Indian	155	96	283	27	181	11	Cases (age: 43.5±9). Controls were age-matched healthy volunteers.	
Makrythanasis P et al.	2010	Greek	202	103	367	37	181	25	Controls (age: 30.7±5.7). Cases (age: 53.3±13.5).	
Kieszko R et al.	2010	Polish	84	128	139	29	199	57	Cases (age: 39.5±11.7, M/F: 60/70). Controls were age-matched healthy volunteers.	
Petković TR et al	2013	Serbian	50	70	75	25	114	26	Controls (age: 47.9±14.2). Cases (age: 49.2±12.8).	
**TNF-α T-1031 C**					T	C	T	C		
Grutters JC et al.	2002	Caucasian	576	196	926	226	322	70	The controls were sex- and age-matched healthy volunteers.	
Sharma S et al.	2008	Indian	155	96	210	100	138	54	Cases (age: 43.5±9). Controls were age-matched healthy volunteers.	
Makrythanasis P et al.	2010	Greek	204	103	323	85	160	46	Controls (age: 30.7±5.7). Cases (age: 53.3±13.5).	
**TNF-α C-863 A**					**C**	**A**	**C**	**A**		
Grutters JC et al.	2002	Caucasian	576	196	973	179	337	55	The controls were sex- and age-matched healthy volunteers.	
Sharma S et al.	2008	Indian	155	96	210	100	138	54	Cases (age: 43.5±9). Controls were age-matched healthy volunteers.	
Makrythanasis P et al.	2010	Greek	192	95	314	70	155	35	Controls (age: 30.7±5.7). Cases (age: 53.3±13.5).	
**TNF-α G-238 A**					**G**	**A**	**G**	**A**		
Yamaguchi E et al.	2001	Japanese	161	110	315	7	212	8	Cases (age: 39.4; M/F: 36/74). Controls were age-matched healthy volunteers.	
Pandey JP et al.	2002	Caucasian	278	278	538	18	533	23	The controls were sex- and age-matched healthy volunteers.	
Pandey JP et al.	2002	African Americans	219	219	419	19	419	19	The controls were sex- and age-matched healthy volunteers.	
Grutters JC et al.	2002	Caucasian	576	196	1108	44	379	13	The controls were sex- and age-matched healthy volunteers.	
Sharma S et al.	2008	Indian	155	96	296	14	185	7	Cases (age: 43.5±9); Controls were age-matched healthy volunteers.	
Makrythanasis P et al.	2010	Greek	209	107	406	12	207	7	Controls (age: 30.7±5.7). Cases (age: 53.3±13.5).	
**TNF-β A252G**					**A**	**G**	**A**	**G**		
Mrazek F et al.	2005	Czechs	425	114	617	233	136	92	Controls (age: 33.8±8.2). Cases (age: 44.8±11.2).	
Sharma S et al.	2008	Indian	155	96	244	66	138	54	Cases (age: 43.5±9). Controls were age-matched healthy volunteers.	
Kieszko R et al.	2010	Polish	84	130	129	39	175	85	Cases (age: 39.5±11.7, M/F: 60/70). Controls were age-matched healthy Volunteers.	

*Abbreviations:* M/F: males/females. The unit for age was years in characteristics.

Of these studies, 12 articles examined the association of the TNF-α gene G-307A polymorphism with sarcoidosis [7-17,19]; five articles focused on the TNF-α gene G-238A polymorphism [10,12,14,15,17]; four articles focused on the TNF-α gene C-857T polymorphism [14,15,17,18]; three articles focused on the TNF-α gene T-1031C and C-863A polymorphisms [14,15,17]; three articles focused on the TNF-β gene A252G polymorphism [13,14, 16].

### Overall analyses

**Figure 2:**
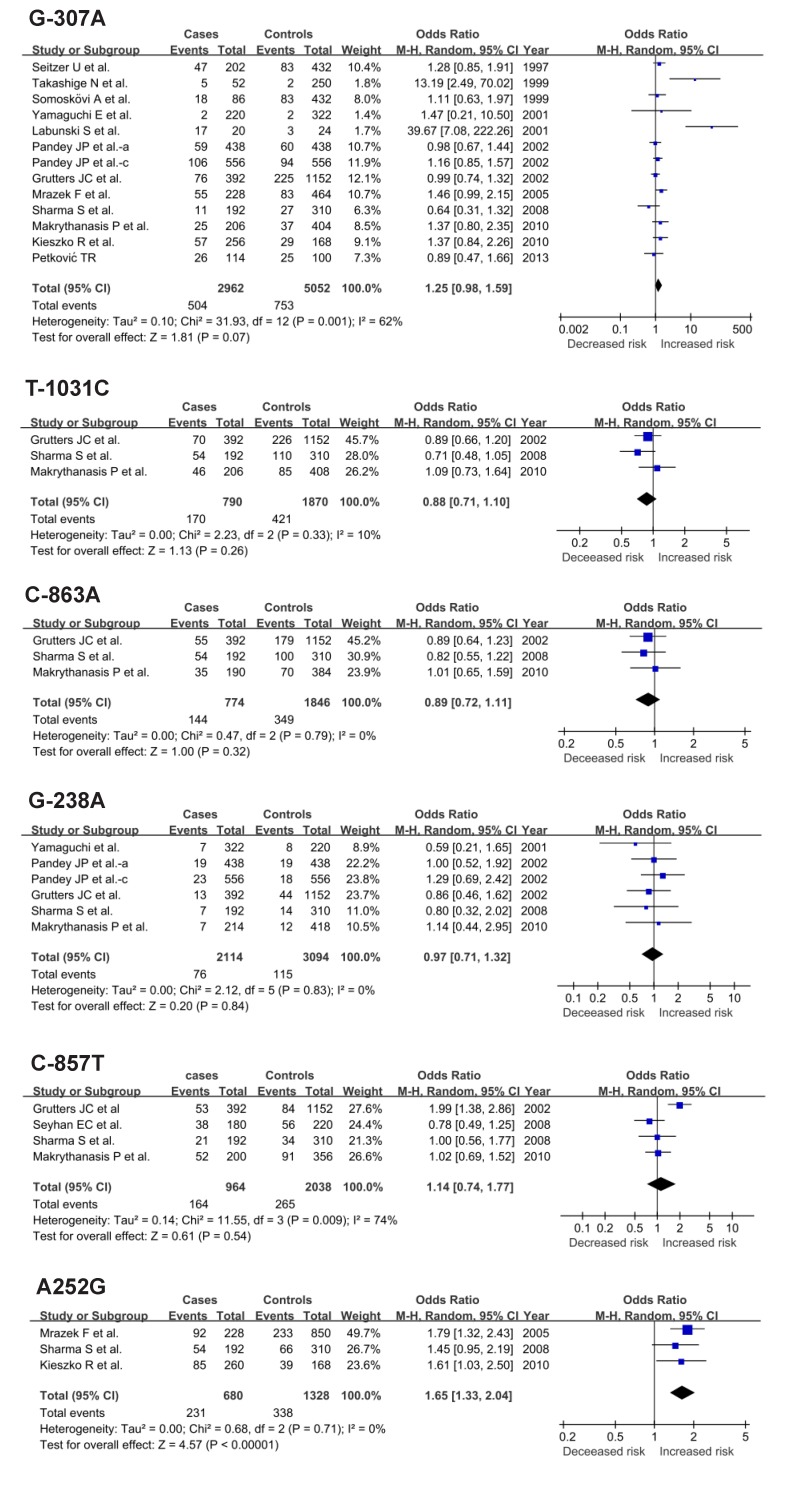
Pooled risk estimates of TNF-α gene G-307A, T-1031C, C-863A, G-238A, C-857T polymorphisms, and TNF-β gene A252G polymorphism in association with sarcoidosis under allelic model.

Figures 2 depicts the pooled risk estimates of developing sarcoidosis for the mutant alleles of the TNF-α and TNF-β gene four polymorphisms. Under a random-effects model, analyses of the full data set failed to reveal any significance for -307A (OR=1.25; 95% CI: 0.98-1.59), -1031C (OR=0.88; 95% CI: 0.71-1.1), -863A (OR=0.89; 95% CI: 0.72-1.11), -238A (OR=0.97; 95% CI: 0.71-1.32), and -857T (OR=1.14; 95% CI: 0.74-1.77) alleles in association with sarcoidosis. In contrast, relative to the TNF-β 252A allele, the risk of having sarcoidosis was 1.65 times (95%CI = 1.33-2.04; P<0.00001) for the TNF-β 252G allele. 

### Subgroup analyses

**Figure 3:**
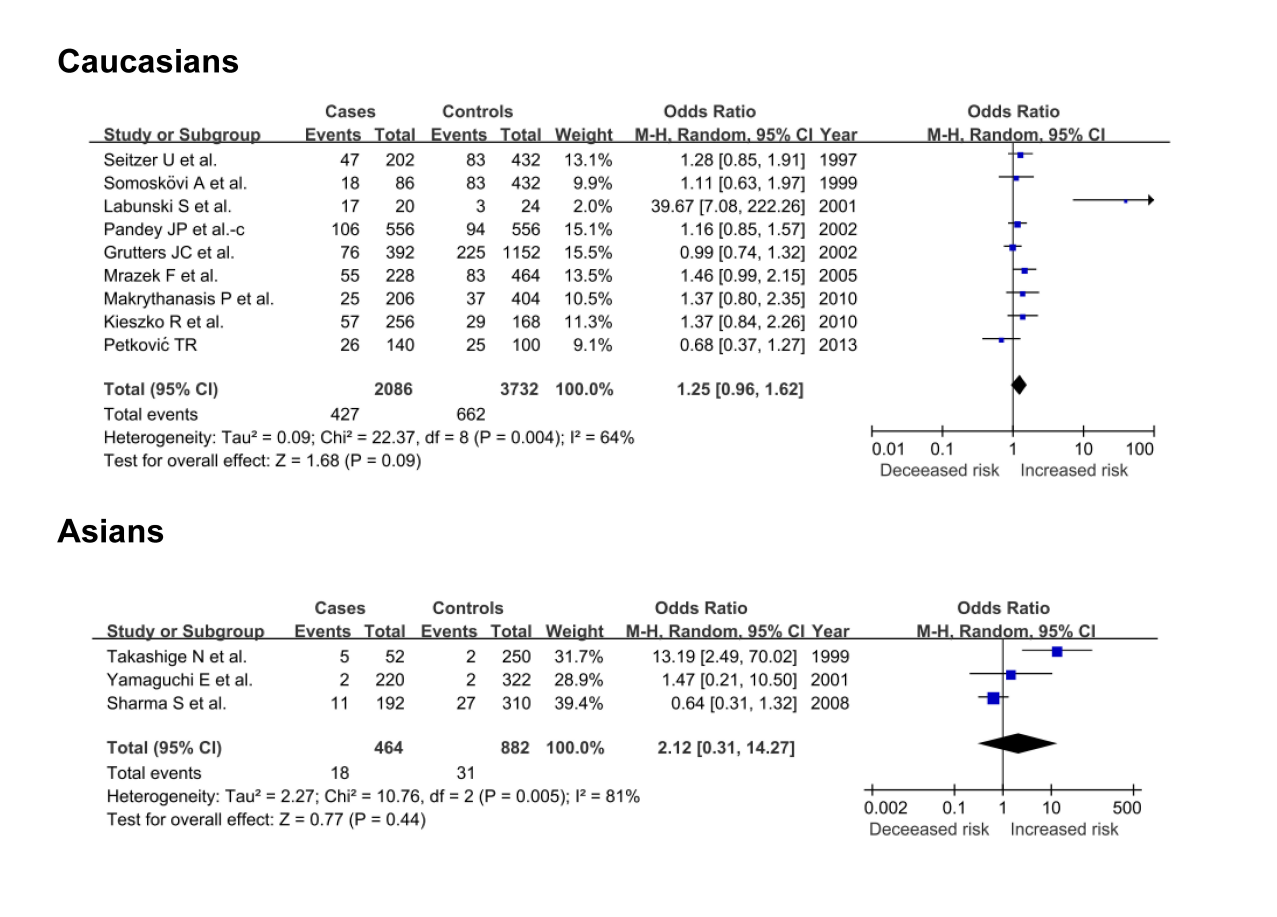
Pooled risk estimates of TNF-α gene G-307A polymorphism in association with sarcoidosis under allelic model by ethnicity.

In view of the number of involved articles, subgroup analyses were only undertaken for TNF-α gene G-307A polymorphism by the ethnicity. Under a random-effects allelic model, there was marginally significant increased risk of sarcoidosis for -307A allele among Caucasians (OR=1.25; 95% CI: 0.96-1.62; P=0.09) but not among Asians (OR=2.12; 95% CI: 0.31-14.27; P=0.44) (Figure 3).

### Publication bias

The *N*_fs_ values were calculated to assess the potential existence of publication bias. At a significant level of 0.05, the *N*_fs0.05_ values were consistently greater than the number of studies included in this meta-analysis for all polymorphisms under investigation.

## Discussion

In this study, we sought to investigate the association of the TNF-α and TNF-β genetic polymorphisms with sarcoidosis risk from English journals by conducting a meta-analysis of 13 articles and 4100 subjects. The principal finding of this study was that the TNF-β gene A252G polymorphism might be a potential risk factor for the development of sarcoidosis. Moreover, subgroup analysis indicated a contributory role of the TNF-α gene G-307A polymorphism in Caucasians. To the authors’ knowledge, this is the first meta-analysis to date investigating the association of the TNF-α gene T-1031C, C-863A, C-857T polymorphisms and the TNF-β gene A252G polymorphism with sarcoidosis.

In 2007, Medica and coworkers have meta-analyzed the TNF-α gene G-307A polymorphism in association with sarcoidosis, and found that this polymorphism was linked to the occurrence of sarcoidosis [20]. Contrastingly by enlarging the sample sizes, we in this meta-analysis failed to replicate the significant finding by Medica and coworkers when pooling all qualified studies together, whereas this significance was somewhat evident after restricting analyses to Caucasian populations, suggesting the genetic heterogeneity of this polymorphisms across ethnic groups. Generally, genetic heterogeneity is an inevitable problem in any disease identification strategy, and populations of different genetic backgrounds may have different linkage disequilibrium patterns. It is reasonable to speculate that the TNF-α gene G-307A polymorphism might be in linkage with another causal variant in one ethnic population but not in another, which was partly exemplified by the wide divergences of the -307A allele frequencies across ethnic populations in this meta-analysis (data not shown). As such, it is necessary to construct a database of susceptible genes and polymorphisms implicated in sarcoidosis in each racial or ethnic group.

Extending previous findings, we provided convincing evidence on the contributory effect of the TNF-β gene A252G polymorphism in the development of sarcoidosis. Although only three articles were available for this association, considering the estimated risk estimate (OR=1.65), it seems unlikely that our results could be explained by confounding. In addition, there was a low probability of publication bias for this comparison as reflected by the fail-safe number. It is reasonable to speculate that, the TNF-β gene A252G polymorphism, if involved, might be implicated in the pathogenesis of sarcoidosis by increasing the production of TNF-β. Large epidemiological and clinical studies are required to fully answer this speculation.

Meta-analysis is recognized as a powerful tool to summarize results of individual studies; however, it is important to recognize certain limitations. First, all qualified studies were retrospective in design, which precludes further comments on causality. Second, albeit a low probability of publication bias in this meta-analysis, potential selection bias cannot be ruled out, because we only retrieved articles published in English language. Third, we selected only five polymorphisms from TNF-α and TNF-β genes, and did not cover other sarcoidosis-susceptibility genes, such as annexin A11 gene [21] and butyrophilin-like 2 gene [22]. Therefore, the jury must refrain from drawing a final conclusion until large, well-designed, prospective studies confirm or refute our findings.

Taken together, this meta-analysis extended previous findings on the association between the TNF-α and TNF-β genetic polymorphisms and sarcoidosis, by showing that the TNF-β gene A252G polymorphism might be a potential risk factor for the development of sarcoidosis. Nevertheless, despite the small sample sizes involved, this meta-analysis provides an anchoring point for better understanding of the pathogenesis of sarcoidosis. For practical reasons, we hope that this study will not remain just another endpoint of research instead of a beginning to establish the background data for further investigation on mechanisms of the TNF-α and TNF-β genes in sarcoidosis.

## Supporting Information

Checklist S1 **PRISMA checklist.**
(DOC)
