# Correction: Association of Six Well-Characterized Polymorphisms in TNF-α and TNF-β Genes with Sarcoidosis: A Meta-Analysis

**DOI:** 10.1371/annotation/679667f2-b4aa-42e8-8c59-511f60256420

**Published:** 2013-12-10

**Authors:** Yun Feng, Jianping Zhou, Chaohao Gu, Yongjie Ding, Huanying Wan, Lei Ni, Wenquan Niu

The third author, Chaohao Gu, was incorrectly indicated as being a Corresponding Author for the article. The third author should instead be indicated as being a first author of the article along with the first two authors, Yun Feng and Jianping Zhou. 

